# Microbial ecology of full-scale wastewater treatment systems in the Polar Arctic Circle: *Archaea*, *Bacteria* and *Fungi*

**DOI:** 10.1038/s41598-018-20633-5

**Published:** 2018-02-02

**Authors:** Alejandro Gonzalez-Martinez, Maija Sihvonen, Barbara Muñoz-Palazon, Alejandro Rodriguez-Sanchez, Anna Mikola, Riku Vahala

**Affiliations:** 1 Department of Built Environment, School of engineering, Aalto University, P.O. Box 15200, Aalto, FI-00076 Espoo Finland; 2 Institute of Water Research, University of Granada, C/Ramón y Cajal, 4, 18071 Granada, Spain

## Abstract

Seven full-scale biological wastewater treatment systems located in the Polar Arctic Circle region in Finland were investigated to determine their *Archaea*, *Bacteria* and *Fungi* community structure, and their relationship with the operational conditions of the bioreactors by the means of quantitative PCR, massive parallel sequencing and multivariate redundancy analysis. The results showed dominance of *Archaea* and *Bacteria* members in the bioreactors. The activated sludge systems showed strong selection of *Bacteria* but not for *Archaea* and *Fungi*, as suggested by diversity analyses. Core OTUs in influent and bioreactors were classified as *Methanobrevibacter*, *Methanosarcina*, *Terrestrial Group Thaumarchaeota* and unclassified *Euryarchaeota* member for *Archaea*; *Trichococcus*, *Leptotrichiaceae* and *Comamonadaceae* family, and *Methylorosula* for *Bacteria* and *Trichosporonaceae* family for *Fungi*. All influents shared core OTUs in all domains, but in bioreactors this did not occur for *Bacteria*. Oligotype structure of core OTUs showed several ubiquitous *Fungi* oligotypes as dominant in sewage and bioreactors. Multivariate redundancy analyses showed that the majority of core OTUs were related to organic matter and nutrients removal. Also, there was evidence of competition among *Archaea* and *Fungi* core OTUs, while all *Bacteria* OTUs were positively correlated among them. The results obtained highlighted interesting features of extremely cold temperature bioreactors.

## Introduction

Undoubtedly, microbial ecology of bioprocesses is a factor of major importance for the functioning of bioprocesses, especially wastewater treatment (WWT) systems^1^. Despite this, in practice, bioprocesses for WWT have been designed from an engineering point of view, but the inherent microbiological aspects of these systems need to be also considered^2^. Prospects in WWT systems design and operation should consider their natural, intrinsic microbiological aspects^3^. Moreover, it has been defended that the understanding of the microbial communities and their activities are essential for the successful exploitation of biological WWT facilities^4,5^.

Activated sludge (AS) systems have been widely used for urban WWT during the last century^6,7^. The AS has been extensively studied, and established and recent results have shown that full-scale AS systems present a core microbiome which activity is responsible for decontamination of wastewater^1,4,7–9^. Nevertheless, the knowledge of microbiome in AS systems is not enough for systems with extreme operational conditions, such as Arctic temperatures^10^.

It has been found that temperature is a parameter that greatly affects microbial community diversity and structure in WWT plants^11^. Cold temperature has been reported to affect microbial growth due to decrease in water availability, molecular motion and energetics, and increase in solute concentration due to decrease in water availability^12,13^. However, the adaptation of microbial communities to low temperature has been found to be problematic for WWT systems. Specifically, biological nitrogen removal processes have a strong negative correlation with temperature, with complete inhibition of mild temperature (around 20 °C) adapted AS when subjected to temperatures of 10 °C or lower^14^. Nevertheless, WWT systems in cold regions obtain high performances in terms of organic matter and nutrients removal. Thus, the microbial communities growing at extremely low temperature in AS systems have adapted to these conditions and an insight of these OTUs would be useful for the exploration of life thriving under cold temperatures and for the design, operation and control of WWT bioprocesses.

In this study, seven full-scale WWT systems, located within the Polar Arctic Circle have been sampled for a characterization of their *Archaea*, *Fungi* and *Bacteria* communities. Quantitative PCR and massive parallel sequencing (MPS) were used for the quantification of microbial OTUs and for the determination of their diversity and relative abundance. Core *Archaea*, *Bacteria* and *Fungi* OTUs were explored for the presence of an oligotype distribution by oligotyping analysis, using for the first time, in full-scale WWTPs studies. Core OTUs were also linked with the operational parameters of the bioreactors in order to unravel their influence over the performance of the system and their susceptibility to temperature. Also, the inter-domain interactions among core OTUs were observed.

## Results and Discussion

### Performance of the bioreactors

The bioreactors analyzed in this study showed an operational temperature ranging from 3 to 7 °C (Table 1). Removal performances for COD, BOD5, Total Nitrogen and Total Phosphorous were of 89.14 ± 10.14%, 93.43 ± 9.78%, 34.57 ± 20.21% and 93.57 ± 8.81%, respectively. The ammonium oxidation efficiency was of 45.43 ± 25.35%. In these cases, the bioreactors were operated with a short sludge age, theoretically not allowing for any significant nitrifying activity due to the fact that the WWT plants didn’t have any requirements for nitrogen removal. Consequently, the observed inefficient nitrification and the absence of denitrifying zones in the systems resulted in low removal performance of total nitrogen. Despite the ultra-low temperature and short sludge retention time, certain WWT systems showed some nitrification probably due to the microbiota adapted to Arctic temperatures.”

**Table 1:** Operational parameters of the full-scale bioreactors analyzed in the study.

WWTPs	Karigasniemi	Kemijärvi	Kolari	Mellanaapa	Rovaniemi	Sirkka	Ylitornio
Code Name	KA	KE	KO	M	R	S	Y
Location	69°23′54″N 25°51′18″E	66°42'48.96″, 27°25′45.12″E	67°19′54.2″ N, 23°47′ 28.73″E	68°39′N, 27°33′E	66°30′N, 025°44′E	67°47′03″N, 24°51′22″E	66°19′10″N, 023°40′15″E
Process Temperature (°C)	3	4	6	5	4	5	7
Air Temperature (°C)	−7.80 ± 0.99	−7.25 ± 1.34	−7.10 ± 1.13	−7.20 ± 0.85	−6.75 ± 1.77	−5.90 ± 1.56	−6.05 ± 1.77
CODinf (mg L^−1^)	906.67 ± 546.02	300.00 ± 95.39	340.00 ± 114.00	1366.67 ± 378.5	543.33 ± 183.39	786.67 ± 124.23	435.00 ± 148.49
BODinf (mg L^−1^)	396.67 ± 228.98	96.33 ± 38.55	94.00 ± 59.00	326.67 ± 128.97	250.00 ± 95.39	300.00 ± 51.96	123.00 ± 66.47
SSinf (mg L^−1^)	506.67 ± 165.0	102.33 ± 24.09	130.00 ± 52.06	1013.33 ± 161.6	126.67 ± 25.17	480.00 ± 147.31	220.00 ± 56.57
Ninf (mg L^−1^)	89.33 ± 41.53	45.00 ± 8.72	46.00 ± 6.00	114.00 ± 19.70	65.00 ± 10.54	71.33 ± 25.01	47.00 ± 9.90
Pinf (mg L^−1^)	10.37 ± 3.73	5.73 ± 1.80	5.30 ± 1.50	21.00 ± 3.46	11.53 ± 3.72	11.83 ± 2.02	6.05 ± 2.19
NH3-Ninf (mg L^−1^)	89.33 ± 41.53	45.00 ± 8.72	46.00 ± 0.15	114.00 ± 19.70	65.00 ± 10.54	71.33 ± 25.01	36.00 ± 7.07
CODeff (mg L^−1^)	37.00 ± 4.00	37.33 ± 2.89	110.00 ± 35.20	38.00 ± 6.56	42.67 ± 4.16	30.00 ± 0.00	56.50 ± 6.36
BODeff (mg L^−1^)	3.00 ± 0.00	5.50 ± 2.41	26.00 ± 8.08	3.83 ± 1.44	5.17 ± 1.01	3.00 ± 0.00	8.05 ± 7.00
SSeff (mg L^−1^)	9.67 ± 3.21	5.23 ± 1.48	48.00 ± 24.33	2.40 ± 0.57	6.23 ± 0.67	5.53 ± 4.73	31.00 ± 21.21
Neff (mg L^−1^)	41.33 ± 7.37	27.33 ± 8.33	46.00 ± 6.00	79.00 ± 20.07	53.00 ± 7.94	48.33 ± 10.79	23.00 ± 5.66
Peff (mg L^−1^)	0.33 ± 0.12	0.25 ± 0.02	1.30 ± 0.7	0.05 ± 0.03	0.14 ± 0.04	0.08 ± 0.03	0.60 ± 0.21
NH3-Neff (mg L^−1^)	39.00 ± 6.93	14.40 ± 6.41	46.00 ± 0.17	53.67 ± 15.82	49.67 ± 8.50	37.33 ± 37.33	10.85 ± 8.70
CODremoval (%)	94.31 ± 4.07	86.91 ± 3.18	68.00 ± 2.11	97.12 ± 0.75	91.68 ± 2.12	96.13 ± 0.56	86.47 ± 3.15
BODremoval (%)	98.90 ± 0.91	93.98 ± 3.03	72.00 ± 0.84	98.70 ± 0.63	97.70 ± 1.10	98.98 ± 0.20	94.14 ± 2.52
SSremoval (%)	96.53 ± 3.80	94.84 ± 1.28	63.00 ± 2.50	99.77 ± 0.01	94.87 ± 1.58	98.77 ± 1.07	86.71 ± 6.22
Nremoval (%)	48.10 ± 18.18	39.62 ± 13.57	0	27.36 ± 31.56	23.67 ± 1.79	28.62 ± 23.27	51.25 ± 1.77
Premoval (%)	96.58 ± 1.42	95.46 ± 1.16	75.00 ± 0.01	99.75 ± 0.09	98.68 ± 0.47	99.33 ± 0.16	90.07 ± 0.09
NH3-Nremoval (%)	51.24 ± 16.49	68.88 ± 8.97	0	53.41 ± 7.01	18.28 ± 3.67	44.85 ± 14.77	66.85 ± 30.67
PE (habitants)	143	2900	914	5957	43371	5486	1920
SRT (d)	n.d.	4	n.d.	n.d.	4	7.3	n.d.
HRT (h)	14	9	n.d.	n.d.	3	15	20
Fe/P	2.5	8.84 ± 5.37	2.90 ± 0.01	n.d.	1.02 ± 0.25	0.80 ± 0.37	8.66 ± 3.14
SVI (mL g^−1^)	n.d.	130	n.d.	n.d.	n.d.	150	n.d.
OLR (Kg-BOD m^−3^ d^−1^)	0.44 ± 0.25	0.20 ± 0.06	0.50 ± 0.01	0.01 ± 0.00*	0.83 ± 0.18	0.30 ± 0.01	0.09 ± 0.02

^*^BOD loading rate per surface area in biorotor; WWTP: WasteWater Treatment Plant; CODinf: Chemical Oxygen Demand of the influent; CODeff: Chemical Oxygen Demand of the effluent; BODinf: Biological Oxygen Demand ad day 5 in the influent; BODeff: Biological Oxygen Demand ad day 5 in the effluent; SSinf: Suspended Solids in the influent; SSeff: Suspended Solids in the effluent; Ninf: Total Nitrogen in the influent; Neff: Total Nitrogen in the effluent; Pinf: Total Phosphorus in the influent; Peff: Total Phosphorus in the effluent; NH3-Ninf: Ammonium measured as Nitrogen in the influent; NH3-Neff: Ammonium measured as Nitrogen in the effluent; CODremoval: Removal rate of COD; BODremoval: Removal rate of BOD; SSremoval: Removal rate of Suspended Solids; Nremoval: Removal rate of Total Nitrogen; Premoval: Removal rate of Total Phosphorus; NH3-Nremoval: Removal rate of Ammonium measured as Nitrogen; PE: Population Equivalents; SRT: Solids Retention Time; HRT: Hydraulic Retention Time; Fe/P: Iron to Phosphorus ratio; SVI: Sludge Volumetric Index; OLR: Organic Loading Rate.

### Number of *Archaea*, *Bacteria* and *Fungi* in the bioreactors sampled in the study

The number of copies of the 16S rRNA gene of *Archaea*, 16S rRNA gene of *Bacteria*, and the 18S rRNA gene of *Fungi* is shown in Figure S1. For *Archaea* only the samples from KA and M had more copies in the influent than inside the bioreactor. This was true for *Bacteria* for samples KA, M and S. On the other hand, the majority of samples had more copies of *Fungi* 18S rRNA gene in the influent than in the bioreactor. Thus, it is possible that *Fungi* could not proliferate under the conditions given in the bioreactors or could not compete with *Archaea* and *Bacteria* OTUs within them.

### Sequence coverage analysis

The results of the redundancy abundance-weighted coverage analysis for all MPS samples used for ecological study of *Archaea*, *Bacteria* and *Fungi* are shown in Table S1. The coverages were very high for the three domains. The required effort ratios for nearly-complete coverage of *Archaea*, *Bacteria* and *Fungi* were relatively low, with none higher than 6%. Therefore, the redundancy abundance-weighted coverage analysis showed that the MPS of *Archaea*, *Bacteria* and *Fungi* was successful.

### α-diversity indices and similarity of samples from the WWT plants analyzed in the study

The α-diversity analysis showed that the three domains suffered a decrease of diversity when the influent wastewater entered the bioreactors sampled (Table S2). These results are different than those obtained from similar full-scale bioreactors in warmer climate conditions, in which diversity and evenness increased inside the bioreactor with respect to the influent wastewater as shown by increase in Shannon-Wiener index and equal values for the Simpson index^7^. Thus, it is possible that cold climate conditions could favor the growth of different OTUs in the sewer system than inside the bioreactor.

The clustering of *Archaea*, *Bacteria* and *Fungi* communities in the influent and mixed liquor of the bioreactors are shown in Figure S2. Thus, the cluster analysis showed that *Bacteria* was the domain presenting the smoother differences within the samples collected. Interestingly, the influent samples showed significant differences among them and were grouped in different groups for *Archaea*, *Bacteria* and *Fungi* domain. In this sense, influent and bioreactor communities for WWTPs KE, M and R were similar for the three domains. This was different that other results found for *Bacteria* in influent wastewater in 10 full-scale WWT plants in The Netherlands and Spain, where influent samples clustered within the same group at the 60% similarity as measured by UniFrac and Bray-Curtis similarity distance, showing a similar *Bacteria* community composition^7^. Also, there was no differentiation in the technological configuration of bioreactors as the Mellanaapa biorotor (sample MB) with respect to the other activated sludge systems. This also contrasts with the results obtained for conventional and A-stage activated sludge, which were well differentiated when operating in warmer climates^7^. It is possible that low temperature or cold climate conditions in general greatly affects diversity in influent wastewater leading to very different compositions within the same geographical location, contrarily as has been reported before^7,8^.

### Microbial ecology of the WWT plants analyzed in the study

**Figure 1:**
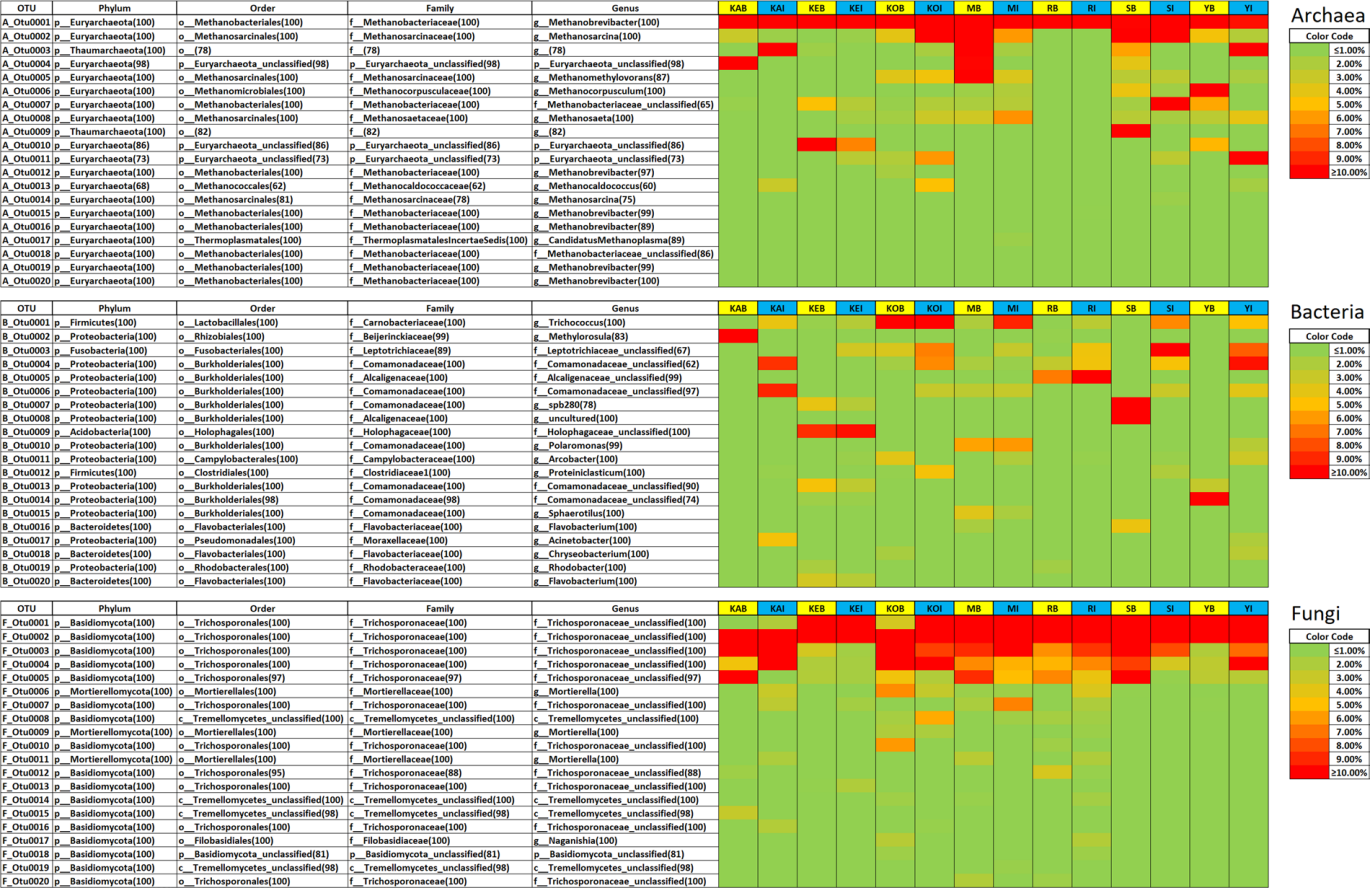
Heat map of the microbial communities in the Influent (I) and Bioreactor (B) samples (A: *Archaea*, B: *Bacteria*, F: *Fungi*).

The community structure of *Archaea*, *Bacteria* and *Fungi* in the bioreactor and influent samples is given in Fig. 1.

#### *Archaea* domain

The dominant phylum among the most important OTUs for the *Archaea* domain belonged to phylum *Euryarchaeaota*. Only two of these most important OTUs belonged to other phyla, namely *Thaumarchaeota*, which account for high relative abundance in samples KAI, MB, SB and YI (5.8–54.9% total relative abundance). The dominant *Archaea* OTU was found at high relative abundance in all samples and was related to *Methanobrevibacter* genus (9.6–70.3%). This is the first time that a cold-adapted *Methanobrevibacter* OTU has been found as ecologically important in bioreactors. Following the criteria established by Gonzalez-Martinez *et al*.^7^ for the definition of core genera, OTUs A_Otu0002 and A_Otu0003 were influent core genera and were classified as *Methanosarcina* and *Terrestrial Group Thaumarchaeota*. Also, OTUs A_Otu0007 was classified as bioreactor core genera and belonged to *Methanobacteriaceae* family. Interestingly, OTU A_Otu0004 had a clear dominance of sample KAB (30.8%) and was identified as an unclassified *Euryarchaeota*.

The structure of the *Archaea* community was defined by members of *Archaea* that have been widely reported in anaerobic digestion processed, such as *Methanobrevibacter*, *Methanosarcina* and *Methanobacteriaceae* family^14^. Besides, the presence of methanogenic *Archaea* in permafrost suggest that these microorganisms are adapted to cold environments^15^. Since none of the bioreactors have anaerobic digestion processes, the presence of methanogenic archaea could be possible due to generation of anaerobic zones within the bioreactors where these organisms can develop their metabolisms.

The terrestrial *Thaumarchaeota* member could possible drive ammonium oxidation, since the majority of ammonium oxidizing archaea found in soil develop this metabolism^16^.

#### *Bacteria* domain

The dominant OTUs of *Bacteria* domain were related to *Trichococcus*, *Methylorosula*, *Polaromonas*, *Arcobacter*, and members of the *Leptotrichiaceae*, *Comamonadaceae*, *Alcaligenaceae* and *Holophagaceae* families. Among these, OTUs B_Otu0001, B_Otu0003 and B_Otu0004 were considered as core OTUs in influent samples. These were affiliated with *Trichococcus* genus, *Leptotrichiaceae* and *Comamonadaceae* families. *Trichococcus* is a common pathogen in sewage^17^, and has been associated with bulking as well as with protein degradation in AS systems at low temperature^18,19^. With respect to Polar environments, facultatively anaerobic *Trichococcus* have been isolated from Arctic tundra soil^20^. The high metabolic versatility of *Trichococcus* may help this genus to adapt to severe temperature conditions such as Polar Arctic Circle WWTPs.

Interestingly, OTU B_Otu0002 had a clear dominance of sample KAB (75.2%) and was classified as *Methylorosula*, which refers to a genus of psychrophilic, chemoorganoheterotrophic, aerobic bacteria^21^. *Methylorosula* was dominant in the bioreactor operating at lower minimum temperature (3 °C), while it was not present in any other influent or bioreactor at relative abundance >2%. This fact may indicate that, among other factors, this genus becomes of importance when no other competitors can adapt to temperatures lower than 4 °C. This is the first time that *Methylorosula* genus has been identified as dominant OTU in a wastewater treatment bioreactor, and thus it is possible that this occurs only at very low operational temperatures. Several uncultured *Burkholderiales* representatives were found as dominant OTUs for samples such as the putative GAO *Comamonadaceae spb280* representative^22^.

No OTUs could be considered as core genera in bioreactors, which contrasts with previous results obtained in full-scale WWTPs in The Netherlands and Spain where core genera were found within bioreactors of the same technological configurations^7^. In this sense, the *Bacteria* community structure in Polar Arctic Circle full-scale WWTPs is not driven by technological configuration as in warmer climates. On the other hand, and as previously reported by Gonzalez-Martinez *et al*.^7^, there were core *Bacteria* OTUs in the influent wastewater. Nevertheless, core genera from different climate zones (The Netherlands and Polar Arctic Circle) were not shared, which indicates that sewage *Bacteria* community structure might be driven by environmental temperature, among other factors.

#### *Fungi* domain

Among the *Fungi* domain, the five most dominant OTUs were affiliated to the *Trichosporonaceae* family and could be classified as core genera in both influent and bioreactor samples according to Gonzalez-Martinez *et al*.^7^ criteria. Genera *Trichosporon* has been reported as arctic-adapted yeast^23^, and the results obtained suggested that *Trichosporonaceae* could play an important role in microbial metabolism in both influents and bioreactors in Polar Arctic Circle full-scale WWTPs, since it is known that *Trichosporon* can denitrify and help *Bacteria* OTUs in nitrogen removal^24^. Besides *Trichosporonaceae* family members, the other OTUs represented were related to *Mortierella* and *Naganishia* genera, which have been isolated from Arctic and Antarctic soil samples, respectively^25,26^.

### Oligotypes of *Archaea*, *Bacteria* and *Fungi* core OTUs in influents and bioreactors sampled in the Polar Arctic Circle regions

**Figure 2:**
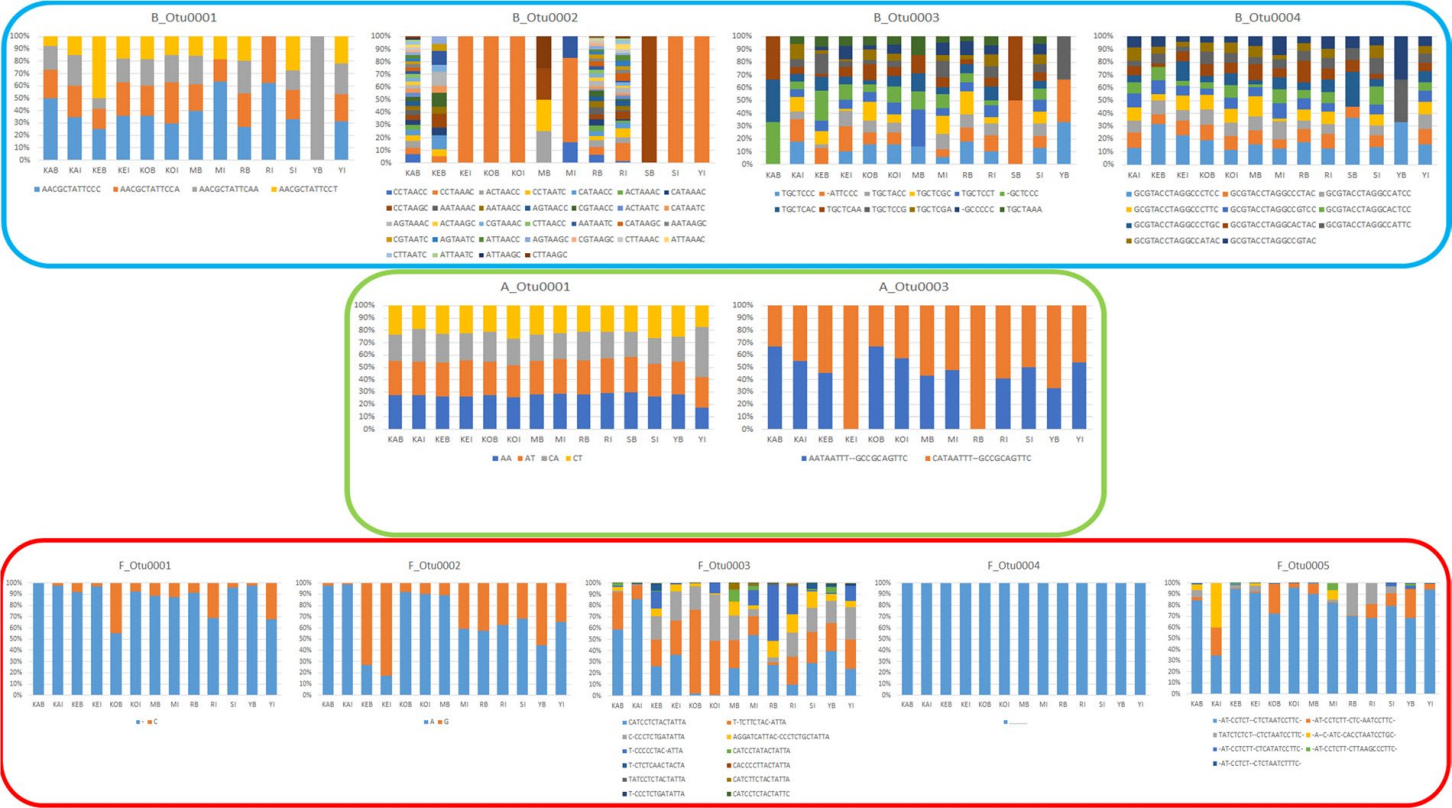
Oligotype structure of OTUs of interest for the *Archaea* (green globe), *Bacteria* (blue globe) and *Fungi* (red globe) domains in the Influent (I) and Bioreactor (B) samples. Each OTU is represented in one graph and accompanied by its relevant oligotypes at the bottom.

All the OTUs that could be classified as core in influent samples, bioreactor samples, both, or those with a clear dominance in a given sample, were taken for an evaluation of their respective oligotypes distribution. Domains *Archaea* and *Bacteria* only accounted for one OTU with a well-expressed oligotype structure (OTUs A_Otu0001 and B_Otu0002), while in contrast, all *Fungi* OTUs of interest had a well-expressed oligotype structure (Table S3 and Table S4). These results showed that *Fungi* OTUs had ubiquitous oligotypes within all influent and bioreactor samples in the Polar Arctic Circle full-scale WWTPs analyzed, while this did not occur for *Archaea* and *Bacteria* (Fig. 2). High relative abundance of *Trichosporonaceae* members to conditions in both sewage and bioreactors highlights its superior adaptation to these conditions in wastewater at cold temperatures.

*Methanobrevibacter*-related OTU A_Otu0001 showed an oligotype distribution with equally represented oligotypes, indicating that no oligotypes of *Methanobrevibacter* had any adaptive advantages within the bioprocesses analyzed. Among *Bacteria* OTUs of interest, it was of importance the high dominance of a *Leptotrichiaceae*-related oligotype of OTU B_Otu0002, which was the only representative in several influent samples. For the *Fungi* domain, it was clear that a certain oligotype dominated all samples for OTUs F_Otu0001, F_Otu0002, F_Otu0004 and F_Otu0005. This evidences that there were ubiquitous oligotypes of *Trichosporonaceae* members in full-scale Polar Arctic Circle WWTPs. The representative sequences on the dominant *Fungi* oligotypes were classified as *Trichosporon sp*., *Cutaneotrichosporon gueohae*, *Trichosporon akiyoshidainum* and uncultured *Tremellomycetes* member (Table S5). In this sense, the results support that *Fungi* OTUs are ubiquitous in full-scale wastewater treatment plants operating in the Polar Arctic Circle while *Bacteria* and *Archaea* seem to have a more local-driven diversity.

Interestingly, the oligotypes present in *Archaea* and *Fungi* domains were much less diverse than for *Bacteria* domain. This may be caused by a better adaptation of *Bacteria* OTUs to Arctic temperatures leading to proliferation of many different oligoytypes within their OTUs; or to the presence of an ecological niche for the dominant OTUs of *Archaea* and *Fungi*, which would displace all other not well-adapted oligotypes to negligible importance within the OTU oligotype structure. More research is required in order to evaluate the role of temperature over the selection of oligotypes of *Archaea*, *Bacteria* and *Fungi* oligotypes in wastewater treatment systems.

### Linkages between operational conditions, microbial diversity, abundance and community structure

#### Linkage between *Archaea*, *Bacteria* and *Fungi* abundance and performance parameters

**Figure 3:**
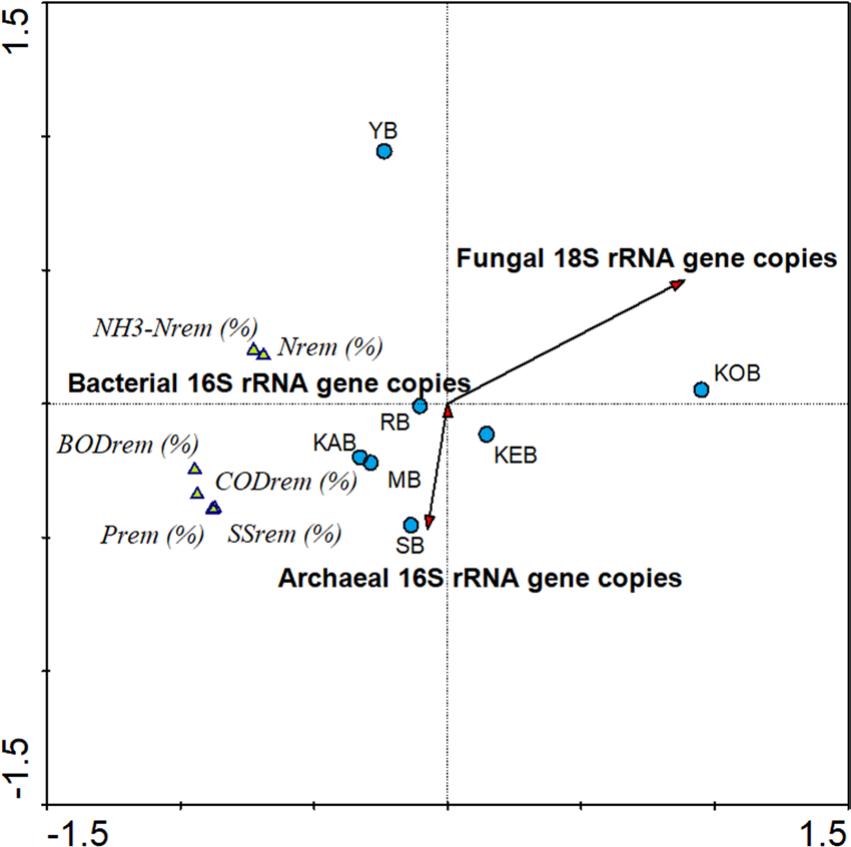
Multivariate redundancy analysis of the copies of ribosomal genes of *Archaea*, *Bacteria* and *Fungi* against the operational parameters in the bioreactors analyzed.

The RDA joining the results of the qPCR with the operational performance of the bioreactors analyzed showed that the copies of *Archaea* 16S rRNA gene are positively correlated with organic matter removal and negatively with ammonia oxidation and nitrogen removal (Fig. 3). The copies of *Bacteria* 16S rRNA gene had a small influence whereas the copies of *Fungi* 18S rRNA gene were negatively correlated with all performance parameters. In this sense, the results suggested that the populations of *Archaea* domain could have a potential effect over the performance of full-scale bioreactors operating in the Polar Arctic Circle region, while *Fungi* would cause an adverse effect.

It has been proposed that *Fungi* cause bulking problems in WWTPs, reducing the settleability of sludge and thus negatively impacting the performance of activated sludge systems^27^. The qPCR measurements for 18S rRNA gene of *Fungi* were related to poor suspended solids removal, matching the three bioreactors with higher *Fungi* gene copies (YB, KOB and KEB) with the lowest performance in solids removal (lower than 63, 85% and 95%, respectively) (Table 1 and Figure S1). The fact that *Fungi* rRNA gene copies were one order of magnitude greater than in other bioreactors coupled with the poor solids removal suggested that *Fungi* in Polar Arctic Circle WWT plants can cause bulking problems and are therefore negative for their performance. On the other hand, the linkage of performance with *Archaea* 16S rRNA gene copies could be attributed to their ecological roles in activated sludge systems. Some studies have pointed out that methanogenic archaea could improve organic matter removal, nitrification and denitrification by syntrophic with members of *Bacteria*, as well as they play an important role in floc formation^28^. Importantly, the high affinity that some *Thaumarchaeaota* OTUs have for oxygen and ammonia could promote their growth over competitor ammonium oxidizing bacteria^29^. In this sense, the presence of methanogenic archaea and terrestrial *Thaumarchaeaota* members in the Polar Arctic Circle WWT plants analyzed suggested that they could potentially have important metabolic roles for the performance of these bioprocesses. The hypothesis of performance-friendly *Archaea* and performance-unfriendly *Fungi* in these systems should be further evaluated.

#### Linkage of *Archaea*, *Bacteria* and *Fungi* core OTUs with performance parameters

**Figure 4:**
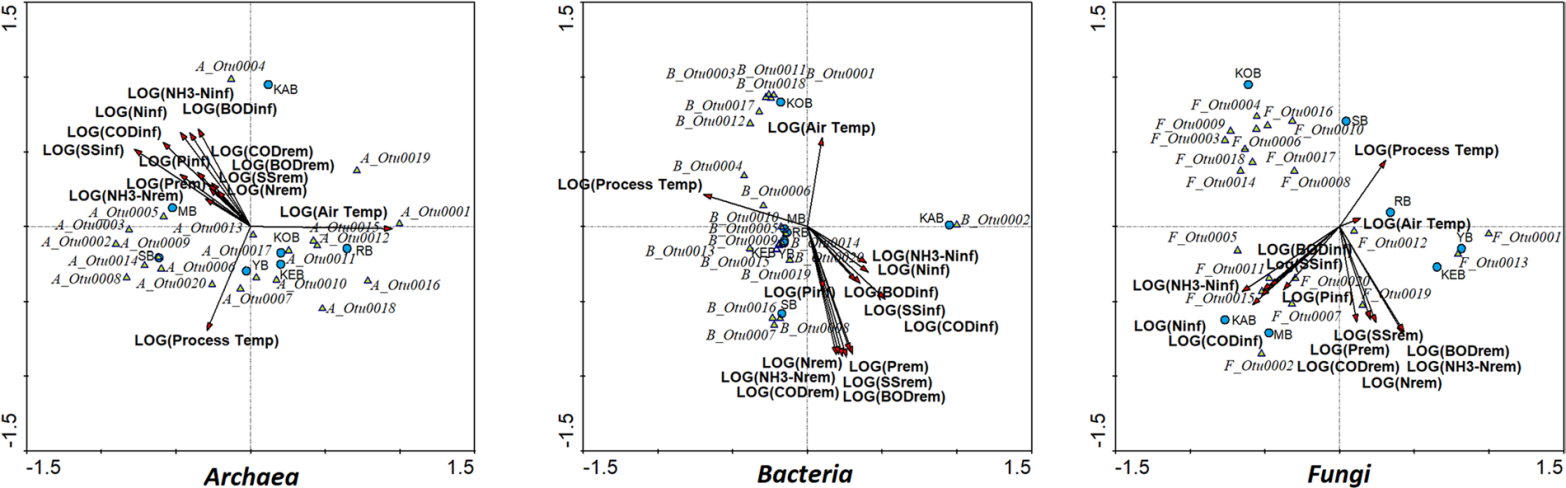
Multivariate redundancy analysis of the dominant OTUs of *Archaea*, *Bacteria* and *Fungi* against the operational parameters, the influent characteristics, and the temperature in the bioreactors analyzed.

The *Archaea* OTUs that were more positively related to performance parameters in the bioreactors were A_Otu0004, A_Otu0003 and A_Otu0002, phylogenetically related to an unclassified *Euryarchaeota* member, a *terrestrial group Thaumarchaeota* OTU and *Methanosarcina* genus, respectively (Fig. 4). The other core OTUs considered for *Archaea* were negatively correlated with performance, with the most important OTU A_Otu0001 being strongly and negatively correlated with bioreactor performance. The strong and positive relationship of A_Otu0003, classified as *Terrestrial Group Thaumarchaeaota*, with removal of ammonia suggested that this OTU might develop ammonium oxidation, therefore standing as the dominant ammonium oxidizing *Archaea* OTU in Polar Arctic Circle full-scale WWTPs. The importance of ammonium oxidizing archaea and *Thaumarchaeaota* on ammonium oxidation in Arctic waters has been highlighted by previous researches^30,31^.

The core *Bacteria* OTUs B_Otu0001, B_Otu0003 and B_Otu0004 were negatively correlated with the performance of the bioreactors (Fig. 4). Only core OTU B_Otu0002 had a positive correlation with removal of organic matter and nutrients, therefore highlighting the potential role that *Methylorosula* and *Beijirenckiaceae* family members could have in Polar Arctic Circle full-scale wastewater treatment plants. The only *Methylorosula* strain that has been isolated, *Methylorosula polaris*, has been found unable to fix nitrogen, unlike other *Beijirenckiaceae* members^32^. The association of *Beijirenckiaceae* members with *Fungi* representatives and *Trichosporon* genus among them has been reported for the formation of membrane biofouling^33^. In this sense, the association of *Beijirenckiaceae* with fungal phylotypes in Polar Arctic Circle WWTPs could be related to formation of biomass at extremely cold operational temperature. The OTUs that had the most positive correlation with performance were B_Otu0007, B_Otu0008 and B_Otu0016, which were taxonomically related with the putative GAO *Comamonadaceae spb280*^22^, an uncultured *Alcaligenaceae* member and *Flavobacterium* genus. Members of the *Alcaligenaceae* family found in full-scale and pilot-scale bioreactors have been identified as denitrifiers and heterotrophic nitrifiers^35–37^. *Flavobacterium* genus was considered a core genus in activated sludge systems in The Netherlands^7^ with floc-forming capabilities. In this sense, it is possible that *Flavobacterium* could develop an important role in wastewater treatment also at lower temperatures.

*Fungi* OTUs F_Otu0002, F_Otu0001 and F_Otu0005 were positively correlated with performance of the bioreactors (Fig. 4). On the other hand, the other core *Fungi* OTUs F_Otu0003 and F_Otu0004 showed a negative correlation. In this sense, *Trichosporonaceae* members found in the bioreactors were not equally contributing to the functioning of the systems. Other important OTUs for the performance were F_Otu0019 and F_Otu0013, which highlighted the strong importance of the *Tremellomycetes* as dominant *Fungi* order in full-scale Polar Arctic Circle bioreactors. The metabolic capabilities of these fungi and their ecological relevance in these systems has yet to be studied. On the other hand, the relationship of *Fungi* and certain bacterial phylotypes could be important in the formation of biomass in these full-scale WWTPs, as it was found of importance in membrane bioreactors^33^.

Since the bioreactors showed deficiencies in terms of ammonium oxidation, a detailed analysis of the OTUs related to ammonia oxidation was conducted. A_Otu0002, A_Otu0003 and A_Otu0005 for *Archaea*; B_Otu0007, B_Otu008 and B_Otu0016 for *Bacteria*; and F_Otu0002, F_Otu0009 and F_Otu0013 for *Fungi*; were the most positively correlated OTUs with ammonia removal. These were classified, respectively, as *Methanosarcina*, terrestrial *Euryarchaeaota*, *Methanomethylovorans*, *Comamonadaceae spb280*, uncultured *Alcaligenaceae* member, *Flavobacterium*, two *Trichosporonaceae* representatives and *Mortierella*. In this way, these OTUs proliferated with increasing ammonia oxidation efficiency, thus they either develop ammonia oxidation or become favored by it. Accordingly, free ammonia nitrogen can inhibit the metabolism of acetotrophic or methylaminotrophic methanogenic archaea, such as *Methanosarcina* and *Methanomethylovorans*, while favoring hydrogenotrophic archaea such as *Methanobrevibacter*^37,38^, explaining the higher relative abundance of A_Otu0001 over A_Otu0002 and A_Otu0005 among other methanogenic archaea found in this study. In this context, the dominant ammonium oxidizing archaea would be the terrestrial *Euryarchaeaota* member. Among *Bacteria*, the metabolisms of *Alcaligenaceae* family and *Flavobacterium* seemed to be more related to denitrification^34–36,39,40^, while *Comamonadaceae spb280* is related to phosphorous removal and therefore no ammonium oxidizing bacteria were found in the bioprocesses^22^. Since neither *Mortierella* nor *Trichosporonaceae* members are known for ammonia oxidation, the results obtained indicated that the dominant ammonia oxidizers are archaea belonging to *Thaumarchaeaota* phylum. In light of this, it is possible that ammonia oxidation in ultra-cold temperature wastewater treatment systems is mainly developed by *Archaea*.

#### Linkage of *Archaea*, *Bacteria* and *Fungi* core OTUs with influent characteristics

The core OTUs in the *Archaea*, *Bacteria* and *Fungi* community structure showed different patterns with respect to influent characteristics, namely concentrations of organic matter, nitrogen, phosphorous and solids (Fig. 4). All these variables were positively correlated among them. The dominant OTU for all domains were strongly and negatively correlated with influent substrate, while the second most important were strongly and positively correlated with them. In this sense, the results suggested that the proliferation of A_Otu0001 over A_Otu0002, B_Otu0001 over B_Otu0002 and F_Otu0001 over F_Otu0002 could be related to the k-strategist behavior of A_Otu0001, B_Otu0001 and F_Otu0001. The strong correlation of core OTUs in all domains, either positive or negative, with influent substrate concentrations indicated that shifts in core OTUs populations could be significantly affected by influent conditions. The trend observed in different behavior of core OTUs with respect to influent conditions was also reported for WWT plants operating under warmer temperatures^7^. In this sense, the operational conditions of the bioreactors analyzed would promote a competition of k-strategists against r-strategists. Thus, *Methanobrevibacter* was found to be a k-strategist in contrast with *Methanosarcina*, the terrestrial *Thaumarchaeota* and the unclassified *Euryarchaeota*. The was true for *Trichococcus* and the representatives of the *Comamonadaceae* and *Leptotrichacaeae* families in contrast with the r-strategist *Methylorosula*. The r-strategist nature of *Methylorosula* may be related with its massive dominance in KAB. In addition, the different *Trichosporonaceae* representative as core *Fungi* OTUs had different behavior with respect to influent conditions, suggesting that shifts in *Trichosporonaceae* communities might be related to influent characteristics.

#### Linkage of *Archaea*, *Bacteria* and *Fungi* core OTUs with temperature

There was a differentiation in the correlation with temperature for the core OTUs of *Archaea*, *Bacteria* and *Fungi* (Fig. 4). OTUs A_Otu0002, A_Otu0003 and A_Otu0004 were strongly and negatively correlated with temperature, as B_Otu0002 and B_Otu0004 for *Bacteria* and F_Otu0002, F_Otu0003 and F_Otu0004 for *Fungi*. On the other hand, OTUs A_0001 and A_Otu0007, B_Otu0001 and B_Otu0003, and F_Otu0001 and F_Otu0004 had a strong positive correlation with temperature.

This could imply several implications for the ecology of *Bacteria*, *Archaea* and *Fungi* in these systems. It seemed that core methanogenic archaea *Methanobrevibacter*, the *Methanobacteriaceae* family member and *Methanosarcina* had different adaptation to temperature, and therefore the two former would displace the last at higher operational temperatures. The OTU related to *Methylorosula* showed a negative correlation with minimum operational temperature, which supports the hypothesis that *Methylorosula* has a competitive advantage at very cold temperature. Among the *Trichosporonaceae* family representatives considered core OTUs, three were more adapted to colder temperature and the other two to warmer temperatures. In this sense, the domination of defined OTUs of *Trichosporonaceae* in the full-scale WWTPs sampled in the Polar Arctic Circle region could be driven by temperature.

#### Linkage among *Archaea*, *Bacteria* and *Fungi* core OTUs

**Figure 5:**
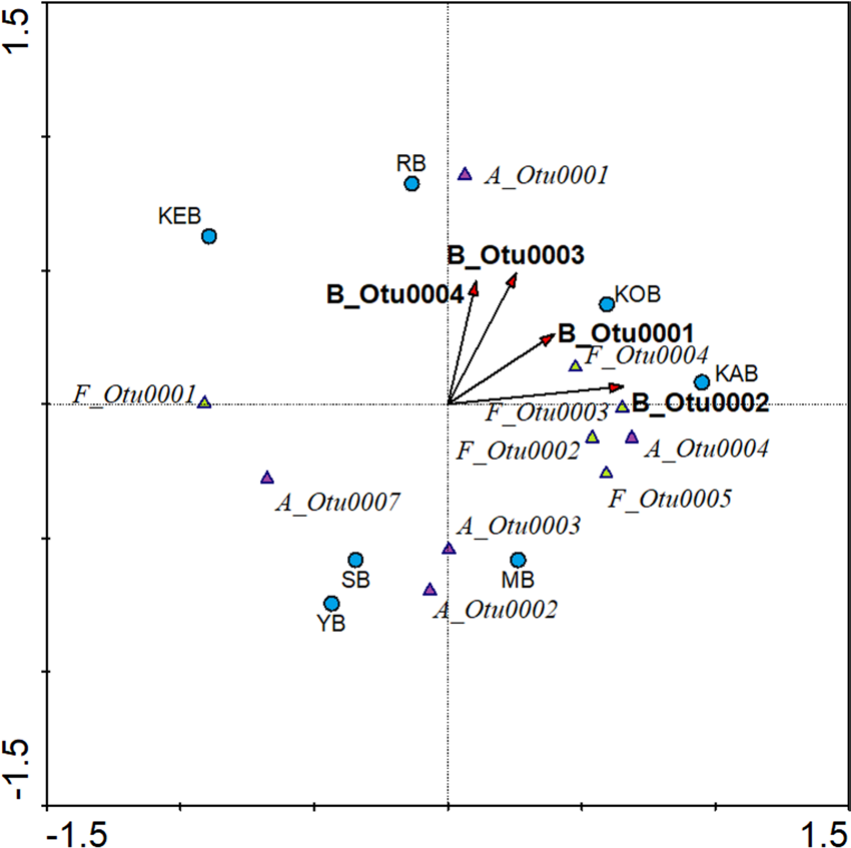
Multivariate redundancy analysis relating the dominant OTUs of *Archaea*, *Bacteria* and *Fungi*.

RDA considering the abundance of the core OTUs for the three domains showed that, while all *Bacteria* core OTUs were relatively related, some of the OTUs for *Archaea* and *Fungi* were antagonists (Fig. 5). Thus, A_Otu0001 appeared in contrast with A_Otu0002, A_Otu0003 and Otu_0007, while A_Otu0004 seemed to be independent of the former OTUs. Interestingly, the antagonism of A_Otu0001, classified as *Methanobrevibacter*, with A_Otu0002 and A_Otu0007, classified as *Methanosarcina* and *Methanobacteriaceae* member, indicated a competition between methanogenic bacteria under Polar Arctic Circle temperatures. The trend seemed similar for *Fungi*, with F_Otu0001 being opposed to all other core *Fungi* OTUs. In this sense, a competition between several OTUs belonging to *Trichosporonaceae* family occurred during the operation of the bioreactors. The negative correlation of F_Otu0001 was also observed with the core *Bacteria* OTUs, which may imply metabolic antagonism with *Bacteria* in the bioreactors analyzed. Thus, results aimed to an interdomain competition between *Bacteria* and *Fungi*. All other core *Fungi* OTUs were positively correlated with *Trichococcus* and *Methylorosula*, as A_Otu0004 which was classified as *Euryarchaeaota* member. The *Leptotrichiaceae* and the *Comamonadaceae* members were strongly, positively correlated with archaeon *Methanobrevibacter*, but not strongly with any *Fungi*.

## Conclusions

Seven full-scale WWTPs located in the Polar Arctic Circle region in Finland were analyzed in order to unravel their microbial community structure in terms of *Archaea*, *Bacteria* and *Fungi*. Results showed that *Fungi* were outcompeted by *Bacteria* and *Archaea* in the bioreactors as shown by qPCR measurements. The microbial communities for the three domains suffered decrease in diversity when entering the bioreactor, as opposed in full-scale WWTPs in warmer climates, and also their structures were more local, in contrast with the similarity presented in influent and bioreactor samples at warmer temperatures, showing the Polar Arctic Circle temperatures were the main factor affecting microorganisms in WWTPs. Core OTUs in influent and bioreactors were found for *Archaea* and *Fungi*, but not for *Bacteria*, contrarily to warmer temperature systems. The main OTUs among *Archaea* were methanogenic archaea, which suffered a competition for dominance between acetotrophic or methylaminotrophic against hydrogenotrophic OTUs. The core *Fungi* OTUs were affiliated with *Trichosporonaceae* family, and their oligotype structure showed a clear domination and ubiquity of certain *Fungi* oligotypes, highlighting their importance in WWT at Polar Arctic Circle temperatures. The composition of *Bacteria* was essentially local and greatly affected by temperature, showing dominance of *Methylorosula*-related microorganisms at minimum temperatures lower than 4 °C. Multivariate redundancy analyses showed that core OTUs were positively correlated with performance of the system and suggesting that terrestrial *Thaumarchaeota* OTU was the main ammonium oxidizing microorganism in the systems. Also, influent characteristics were important for microbial structure dynamics, selecting for dominant k-strategists OTUs. Besides, antagonistic relationships between core *Archaea* and *Fungi* OTUs were observed while core *Bacteria* OTUs were positively correlated among them.

## Materials and Methods

### Selected WWT plants

The full-scale WWT plants sampled in this study were in Arctic climate zones. The samples were collected in late November, when the systems are subjected to extremely low temperature conditions. Seven full-scale bioreactors were analyzed in the study. A summary of the operational conditions of these bioreactors is given in Table 1, and a description of them is given in the Supplementary material.

### Collection of samples and extraction of environmental DNA

The samples from the WWT plants were collected according to the protocol described in Gonzalez-Martinez *et al*.^7^. Briefly, 1000 mL of mixed liquor were taken from a point in the bioreactor where mixing conditions were optimal for each of the WWT plants evaluated in the study. Influent samples were taken as a sub-sample of the time- or flow-proportional 24 h-composite sample. The samples were kept at 4 °C until they reached the laboratory. Then, mixed liquor samples were centrifuged at 3500 rpm during 10 minutes at room temperature for separation of biomass and water. The pelleted biomass was kept at −20 °C until subsequent DNA extraction procedure.

The DNA extraction was done using the FastDNA SPIN Kit for Soil (MP Biomedicals, Solon, OH, USA) and the FastPrep apparatus following the instructions given by the manufacturer. The five biomass samples from the same bioreactor or influent flow yielded five different DNA extracts which were finally merged into the same DNA pool, as has been done before^7^. The DNA pools were then kept at −20 °C and sent to Research and Testing Laboratory for further MPS process.

### Illumina MiSeq MPS

The DNA pools were subjected to MPS procedure using the Illumina MiSeq apparatus and the Illumina MiSeq Reagent v3. This protocol was done three times for each DNA pool to independently identify *Bacteria*, *Archaea* and *Fungi* OTUs. The primer pairs 28FF-519R (5′-GAGTTTGATCNTGGCTCAG-3′ and 5′-GTNTTACNGCGGCKGCTG-3′)^7^, 519F-1041R (5′-CAGCMGCCGCGGTAA-3′ and 5′-GGCCATGCACCWCCTCTC-3′)^14^ and ITS1F-ITS2 (5′-CTTGGTCATTTAGAGGAAGTAA-3′ and 5′-GCTGCGTTCTTCATCGATGC-3′)^24^ were chosen for the amplification of the hypervariable regions V1-V3 of 16S rRNA gene of *Bacteria*, the hypervariable regions V4-V6 of 16S rRNA gene of *Archaea*, and ITS region of *Fungi*, respectively.

### MPS post-process and ecological analysis

The treatment of raw data from MPS was done for *Bacteria*, *Archaea* and *Fungi* using mothur^41^ and VSEARCH^42^ software. First, MiDAS S123 2.1.3 release^43^ was aligned against SiLVA NR v128 release using mothur, and the UNITEv6 database^44^ was aligned against itself using MUSCLE algorithm^45^. MiDAS database was used for the processing of *Bacteria* and *Archaea* sequences, while UNITE database was used for *Fungi*.

For the treatment or raw data, paired-end reads were merged into contigs avoiding the generation of ambiguous bases in the overlap region following Unno *et al*.^46^. The contigs were then screened to eliminate those with >0 ambiguous bases and >8 homopolymers.

The remaining contigs were then aligned against the database of choice using the Needleman criteria. Then, contigs that did not align at the position of the forward and reverse primers were considered as alignment failures and removed from the analysis. The remanent contigs were then subjected to chimera slaying process using VSEARCH. The remaining contigs were then taxonomically affiliated using the database of choice by the k-nearest-neighbor method and searching algorithm using k-mer size of 8. Those that failed to affiliate with their respective domain were removed. Then, all remaining sequences were clustered into OTUs in a 95% similarity threshold for *Archaea* and *Fungi* and 97% similarity threshold for *Bacteria*, respectively, using distance-based greedy clustering method^47,48^ implemented in VSEARCH.

### Quantitative PCR of *Archaea*, *Bacteria* and *Fungi*

The number of copies of *Bacteria* and *Archaea* 16S rRNA and *Fungi* 18S rRNA gene of each of the extracted DNA pools was measured by the means of quantitative real time PCR (qPCR). qPCR was performed using an Mx3000P QPCR system (Agilent Technologies) and the primers and annealing conditions described by Muyzer *et al*.^49^, Yu *et al*.^50^ and Nishizawa *et al*.^51^, for *Bacteria*, *Archaea* and *Fungi*, respectively. All quantitative amplifications were performed in duplicate. qPCR calibration curves were constructed with the aid of plasmid standards harboring inserts of the targeted genes. The calibration curves for the absolute quantification in the DNA samples were generated using serial tenfold dilutions (10^−2^–10^−8^) of linearized plasmid standards. The reaction mixture was made in a total volume of 25 μL contained 0.125 μL of SYBR Green PCR, 2.5 μL of buffer, 1.5 μL of MgCl2, 0.5 μL of dNTPs, 0.15 μL of each primer (10 µM), 0.125 μL of Taq Polymerase, 0.0625 μL of BSA, 17.88 of MilliQ water and 2 μL template DNA diluted 1:10 and 1:50. Melting curves were used at the end of each qPCR to check amplification specificity and purity of negative controls. Real-time PCR data were analyzed using a MxPro QPCR software version 3.0 (Stratagene, USA).

### Sequencing coverage analysis

The MPS samples used for the determination of ecology of *Archaea*, *Bacteria* and *Fungi* were checked for diversity coverage using a redundancy abundance-weighted coverage method. This was done using NonPareil software^52,53^. The calculations were done using a query set size of 1000 sequences and a 95% similarity threshold in a minimum 50% sequences overlap.

### α-diversity indices and similarity analysis of MPS samples

The OTU ecology of the MPS samples were used for the calculation of α-diversity indices Shannon-Wiener and Simpson, and a clustering of samples computed through Bray-Curtis distances. This was done using mothur software.

### Oligotyping analyses of OTUs of interest

Several OTUs were selected for the *Archaea*, *Bacteria* and *Fungi* domains, based on their presence (>1% relative abundance in at least all influent samples, or all bioreactor samples) or high dominance (>25% relative abundance in at least one sample). These OTUs were subjected to an oligotyping analysis following the procedure described by Meren *et al*.^54^. First, Shannon entropy was calculated for each of the OTUs. Based on these results, oligotypes were constructed for each OTU by repeated calculation until the purity score of the oligotypes with >100 reads was >0.90^55^. Removal of noise during the oligotyping process was set as: (i) each oligotype had to appear in at least one sample; (ii) each oligotype had to account for at least 1% relative abundance in at least one sample; (iii) each oligotype had a substantive abundance of 30^56^.

### Multivariate redundancy analyses

Several multivariate redundancy analyses were developed to link: (i) the operational conditions with the number of copies of *Archaea*, *Bacteria* and *Fungi* members; (ii) the performance parameters of the bioreactors and their dominant *Archaea*, *Bacteria* and *Fungi* OTUs; (iii) the influent concentrations of organic matter, nutrients and solids and their dominant *Archaea*, *Bacteria* and *Fungi* OTUs; (iv) the temperature parameters of the bioreactors and the dominant archaea, *Bacteria* and *Fungi* OTUs; (v) the dominant OTUs across different domains (*Archaea* and *Bacteria*, *Archaea* and *Fungi*, *Bacteria* and *Fungi*). All multivariate redundancy analyses were developed using the CANOCO 4.5 for Windows and calculated by 499 unconstrained Monte-Carlo simulations in a full permutation model. Variable with units not consistent represented in the same multivariate redundancy analysis were normalized under a logarithmic model as in Gonzalez-Martinez *et al*.^7^.

## Electronic supplementary material

Supplementary material
