# Supplementary material for: Microbial ecology of full-scale wastewater treatment systems in the Polar Arctic Circle: Archaea, Bacteria and Fungi
Source: Sci Rep. 2018 Feb 2;8:2208. doi: 10.1038/s41598-018-20633-5 (PMC5797233; doi:10.1038/s41598-018-20633-5)
Supplement: Supplementary file 1 — Supplementary material [file 41598_2018_20633_MOESM1_ESM.pdf]

Microbial ecology of full-scale wastewater treatment systems in the Polar Arctic Circle: *Archaea*, *Bacteria* and *Fungi*

Alejandro Gonzalez-Martinez[a], Maija Sihvonen[a], Barbara Muñoz-Palazon[b], Alejandro Rodriguez-Sanchez[b], Anna Mikola[a] , Riku Vahala[a]

[a]: Department of Built Environment, School of engineering, Aalto University, P.O. Box 15200, Aalto, FI-00076 Espoo, Finland.

[b]: Institute of Water Research, University of Granada, C/Ramón y Cajal, 4, 18071 Granada, Spain.

* Corresponding author: Dr. Alejandro Gonzalez-Martinez, Department of Built Environment, Aalto University, P.O. Box 15200, Aalto, FI-00076 Espoo, Finland; E-mail: Alejandro.gonzalezmartinez@aalto.fi;

Polar Arctic Circle Wastewater treatment plants descriptions

Ivalo

Mellanaapa WWTP is a biorotor plant located near the village of Ivalo. It was taken into use in 2005. Population equivalent (PE) was 5957 in 2016. Mellanaapa treats wastewater from Ivalo and Saariselkä, which is a popular ski resort during wintertime. Treated water is first discharged to River Akujoki, from where it flows to Lake Inarijärvi. Ivalo has removal efficiency requirement for BOD_7 and total phosphorus, but no nitrogen removal obligation.

Karigasniemi

Karigasniemi is a small activated sludge (AS) plant located in the village of Karigasniemi. PE in 2016 was 143 and ca 280 inhabitants are connected to the sewer network. Treated wastewater is discharged to River Inarijoki. Karigasniemi has removal requirements for BOD_7, COD_{Cr}, suspended solids and total phosphorus, but no nitrogen removal obligation.

Kemijärvi

Kemijärvi is an AS plant with simultaneous phosphorus precipitation located in the city of Kemijärvi. PE was 2900 in 2016. Treated wastewater is discharged to Lake Kemijärvi. Kemijärvi has removal efficiency requirement for BOD_7 and total phosphorus, but no nitrogen removal obligation.

Kolari

Kolari AS plant was built in 1975 and is located in the village of Kolari. PE in 2016 was 914. Treated wastewater is discharged to River Muonionjoki. Kolari has removal requirement for BOD_7 and total phosphorus, but no nitrogen removal obligation.

Levi

Taalovaara AS plant located near Levi ski resort was built in 1992 and treats wastewater from Kittilä and Levi. In 2016, PE was 5486. The treatment process includes flow equalization basin before AS process. Treated wastewater is discharged to River Ounasjoki. Levi has removal

requirements for BOD_7, COD_{Cr}, suspended solids and total phosphorus, but no nitrogen removal obligation.

Ylitornio

Ylitornio AS plant was taken into use in 1990 and is located in the village of Ylitornio. PE in 2016 was 1920. Treated wastewater is discharged to River Tornionjoki. Ylitornio has removal requirement for BOD_7 and total phosphorus, but no nitrogen removal obligation.

Rovaniemi

Alakorkalo AS plant was built in 1979 and is located in the city of Rovaniemi. PE in 2016 was 43 371. Alakorkalo treats wastewater both from households and industrial sources, such as a peat plant, hospital, concrete factory, laundry, bakeries, plastic film factory and slaughterhouse. Treated wastewater is discharged to River Kemijoki. Rovaniemi has removal requirement for BOD_7 and total phosphorus, but no nitrogen removal obligation.

City/village	Ivalo	Karigas-niemi	Kemi-järvi	Kolari	Levi	Yli-tornio	Rova-niemi
WWTPs	Mellan-aapa	Karigas-niemi	Kemijärvi	Kolari	Taalo-vaara	Yli-tornio	Ala-korkalo
Pre-treatment (Screening and grit removal)	x	x	x	x	x	x	x
Chemically enhanced primary clarification	x	x	-	-	x	x	x
Secondary treatment	x[b]	x[a]	x[a,c]	x[a,c]	x[a]	x[a]	x[a]
Tertiary treatment	x[d]	-	-	-	x[e]	-	-

x Process present

- Process not present

[a] CAS; [b] biorotors; [c] simultaneous precipitation; [d] Coagulation, flocculation and clarification;[e] DAF + UV

Table S1 – Redundancy abundance-weighted coverage analysis of the MPS subsamples

Archaea				Bacteria				Fungi			
Coverage (%)	Actual effort (Mbp)	Required effort (Mbp)	Effort ratio (%)	Coverage (%)	Actual effort (Mbp)	Required effort (Mbp)	Effort ratio (%)	Coverage (%)	Actual effort (Mbp)	Required effort (Mbp)	Effort ratio (%)
98.44	85.59	4.435	5.18	99.37	484.7	20.71	4.27	99.65	132.2	4.10	5.426

Actual effort: base pairs actually sequenced

Required effort: estimated base pairs required for 100% coverage

Effort ratio: Ratio between Required effort and Actual effort.

Table S2 – α-diversity indices of the MSP samples

		Archaea		Bacteria		Fungi	
	Sample	Shannon-Wiener	Simpson	Shannon-Wiener	Simpson	Shannon-Wiener	Simpson
Influent	KAI	2.6246	0.3186	6.3436	0.0217	1.7257	0.2746
	KEI	3.5808	0.2133	6.7893	0.0140	1.3217	0.3880
	KOI	4.0786	0.0633	5.6023	0.0762	2.0576	0.1916
	MI	5.3479	0.0618	7.0762	0.0152	1.9685	0.2175
	RI	2.9756	0.4080	5.4050	0.0849	2.2900	0.1838
	SI	4.4317	0.0638	6.0932	0.0468	1.3555	0.4648
	YI	3.3844	0.1191	5.5660	0.0253	1.6933	0.2679
Bioreactor	KAB	2.4336	0.2847	2.2767	0.5663	1.8369	0.2942
	KEB	3.1615	0.2266	5.9405	0.0175	1.3103	0.4346
	KOB	3.5332	0.2278	6.1592	0.0689	2.3298	0.1565
	MB	3.6062	0.0920	6.7684	0.0094	2.0328	0.2516
	RB	2.5608	0.4939	6.3069	0.0108	2.0560	0.2504
	SB	4.0546	0.0835	5.5865	0.0565	2.1686	0.1846
	YB	3.7427	0.1403	6.4560	0.0184	1.1347	0.5026
Influent	Mean I	3.7748	0.1783	6.1251	0.0406	1.7732	0.2840
	St. Dev. I	0.9265	0.1397	0.6454	0.0295	0.3590	0.1056
Bioreactor	Mean B	3.2989	0.2213	5.6420	0.1068	1.8384	0.2964
	St. Dev.B	0.6096	0.1418	1.5307	0.2040	0.4490	0.1277

Table S3 – Parameters of the oligotypes for OTUs of interest within the *Archaea*, *Bacteria* and *Fungi* domains

OTU	Domain	Total reads	Analyzed reads (%)	Oligotypes	Total purity score
A_Otu0001	Archaea	20438	98.28%	4	0.91
A_Otu0002	Archaea	No oligotypes found			
A_Otu0003	Archaea	2352	37.20%	2	0.96
A_Otu0004	Archaea	No oligotypes found			
A_Otu0007	Archaea	No oligotypes found			
B_Otu0001	Bacteria	15200	5.41%	4	0.93
B_Otu0002	Bacteria	14998	93.05%	32	0.94
B_Otu0003	Bacteria	9349	17.02%	12	0.92
B_Otu0004	Bacteria	8394	19.23%	11	0.92
F_Otu0001	Fungi	134486	99.88%	2	0.97
F_Otu0002	Fungi	100491	99.95%	2	0.90
F_Otu0003	Fungi	36893	90.37%	13	0.93
F_Otu0004	Fungi	32383	93.43%	1	0.99
F_Otu0005	Fungi	13354	88.20%	8	0.97

Table S4 – Definition of dominant oligotypes and second-dominant oligotypes for dominant OTUs

OTU	Main Oligotype	Average Relative abundance	Environment-specific	Competing Oligotype	Average Relative Abundance	Environment-specific
A_Otu0001	AT	27.35762003	Present in all samples	AA	26.81679611	Present in all samples
A_Otu0002						
A_Otu0003	CATAATTT--GCCGCAGTTC	56.88018139	Present in KEI and RB as unique oligotype	AATAATTT--GCCGCAGTTC	43.11981861	
A_Otu0004						
A_Otu0007						
B_Otu0001	AACGCTATTCCC	36.05878185	Not present in YB	AACGCTATTCAA	23.4898153	Unique oligotype in YB
B_Otu0002	CCTAAAC	49.83444929	MB and SB	CCTAATC	3.825256929	Present only in KAB, KEB, RI and RB
B_Otu0003	-ATTCCC	14.02511325	Not present in KAB and MB	TGCTCCC	12.24903188	Not present in KAB, KEB and SB
B_Otu0004	GCGTACCTAGGCCCTCC	19.69362704	Present in all samples	GCGTACCTAGGCCCTAC	9.57456824	Not present in YB
F_Otu0001	-	85.96021928	Present in all samples	C	14.03978072	Not present in KAB
F_Otu0002	A	68.38823103	Present in all samples	G	31.61176897	Present in all samples
F_Otu0003	CATCCTCTACTATTA	30.46820217	Present in all samples	T-TCTTCTAC-ATTA	27.2847793	Present in all samples
F_Otu0004	....	100	Present in all samples			
F_Otu0005	-AT-CCTCT--CTCTAATCCTTC-	76.385181	Present in all samples	-AT-CCTCTT-CTC-AATCCTTC-	10.74187516	Present in all samples

Table S5 – Best BLAST hits of representative sequences of dominant fungal oligotypes against the NCBI nt database

		Best BLAST hit against NCBI nt database				
OTU	Oligotype	Accesion number	Taxonomy	Query cover (%)	e-value	Identity (%)
F_Otu0001	-	FM178264	Uncultured *Tremellomycetes* clone aww38	100	3e-88	98
F_Otu0002	A	LT623974	*Trichosporon sp.* SF19-2	100	3e-82	100
F_Otu0004	.	KX859640	*Cutaneotrichosporon guehoae* isolate URFM545	100	1e-97	100
F_Otu0005	-AT-CCTCT--CTCTAATCCTTC-	KX302021	*Trichosporon akiyoshidainum* isolate M8	100	5e-80	100

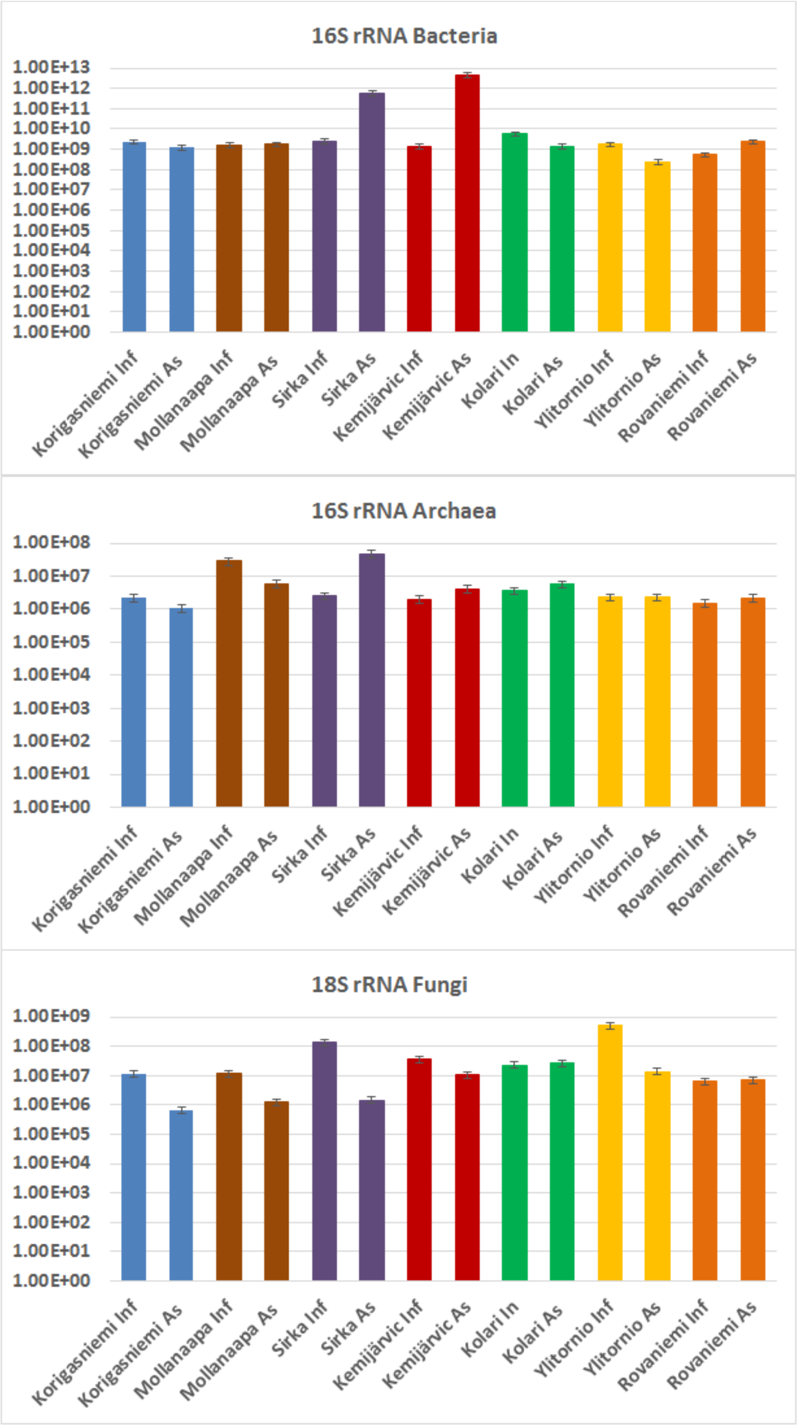

Figure S1 – Amplifications plots of real time qPCR of *Archaea* 16S rRNA gene, *Bacteria* 16S rRNA gene and *Fungi* 18S rRNA gene samples. Dilution 1:10.

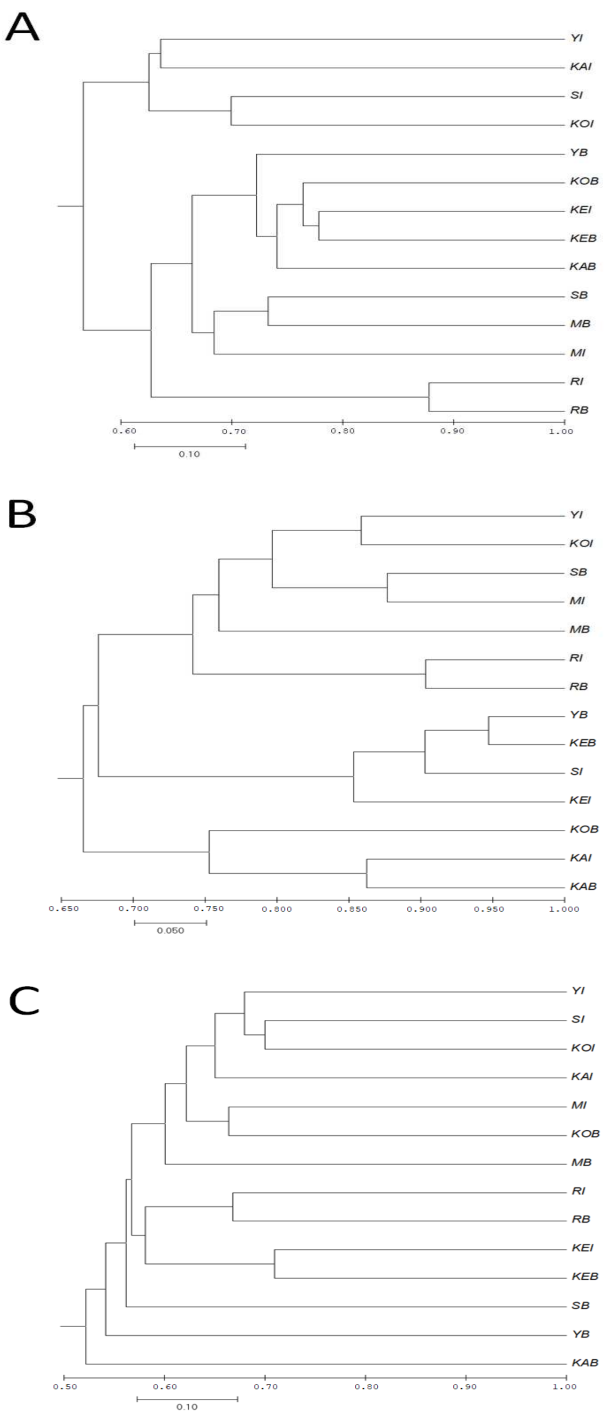

Figure S2 – Bray-Curtis similarity analysis of the Influent (I) and Bioreactor (B) samples (A: Archaea; B: Bacteria; C: Fungi). The last letter of samples names identifies them as influent (I) or bioreactor (B)
